# The Cancer Clock Is (Not) Ticking: Links between Circadian Rhythms and Cancer

**DOI:** 10.3390/clockssleep1040034

**Published:** 2019-09-20

**Authors:** Monica N. Morgan, Sapir Dvuchbabny, Chloe-Anne Martinez, Bernadette Kerr, Peter A. Cistulli, Kristina M. Cook

**Affiliations:** Charles Perkins Centre, University of Sydney, Sydney, NSW 2050, Australia; mmor8146@uni.sydney.edu.au (M.N.M.); sdvu7396@uni.sydney.edu.au (S.D.); cmar9054@uni.sydney.edu.au (C.-A.M.); bernadette.kerr@sydney.edu.au (B.K.); peter.cistulli@sydney.edu.au (P.A.C.)

**Keywords:** circadian rhythms, cancer, clock, circadian disruption, hypoxia, tumor

## Abstract

Circadian rhythms regulate many physiological and behavioral processes, including sleep, metabolism and cell division, which have a 24-h oscillation pattern. Rhythmicity is generated by a transcriptional–translational feedback loop in individual cells, which are synchronized by the central pacemaker in the brain and external cues. Epidemiological and clinical studies indicate that disruption of these rhythms can increase both tumorigenesis and cancer progression. Environmental changes (shift work, jet lag, exposure to light at night), mutations in circadian regulating genes, and changes to clock gene expression are recognized forms of disruption and are associated with cancer risk and/or cancer progression. Experimental data in animals and cell cultures further supports the role of the cellular circadian clock in coordinating cell division and DNA repair, and disrupted cellular clocks accelerate cancer cell growth. This review will summarize studies linking circadian disruption to cancer biology and explore how such disruptions may be further altered by common characteristics of tumors including hypoxia and acidosis. We will highlight how circadian rhythms might be exploited for cancer drug development, including how delivery of current chemotherapies may be enhanced using chronotherapy. Understanding the role of circadian rhythms in carcinogenesis and tumor progression will enable us to better understand causes of cancer and how to treat them.

## 1. Introduction to Circadian Rhythms and Cancer

Cancer is a common cause of death and there is an urgent need to discover both novel causes and treatments. An emerging area of interest is the impact of circadian rhythms on cancer incidence and progression. The pathways that regulate tumor cell circadian rhythms also present new drug target opportunities. Much of mammalian behavior and physiology follows a daily oscillatory circadian rhythm due to adaptation to the Earth’s rotation. The circadian clock controls ~24-h rhythmic homeostasis in body temperature, hormone release, eating and sleeping [1]. On a cellular level, circadian rhythms can regulate cancer-related processes such as cell division, apoptosis and DNA repair. Up to 50% of the genome is thought to be under circadian control, including genes involved in metabolism, cell cycle and growth factors [2,3,4,5,6,7,8,9]. Given the high number of cancer-related genes that are regulated by circadian oscillations, it is hypothesized that dysregulation of the circadian rhythm may play a role in tumorigenesis and/or tumor progression.

Both clinical and laboratory-based studies have suggested links between tumorigenesis and circadian clocks [10,11], but the mechanisms are poorly understood. Environmental circadian disruption through shift work or exposure to light during nighttime has been linked to hormonal cancers such as breast and prostate [12,13,14,15], while genetic disruption of circadian oscillation in animals promotes lung tumorigenesis and progression [10]. Tumor cells themselves have been found to have mutations in clock-related genes [16], and microenvironmental changes caused by growing tumors may disrupt circadian rhythms in surrounding cells [17]. The cellular clock phenotype can vary between cells in the same tumor and between different types of tumors. The mechanisms of circadian disruption in cancers are complicated and have several layers. There is much to be learned in how circadian rhythms can affect both tumorigenesis and/or progression of a pre-existing tumor. This review will focus on the links between cancer and environmental and genetic disruption of circadian rhythms. We will further explore how changes to the tumor microenvironment, such as hypoxia and acidity, can impact on the rhythms within the tumor cells themselves.

### 1.1. Central and Peripheral Clocks

**Figure 1:**
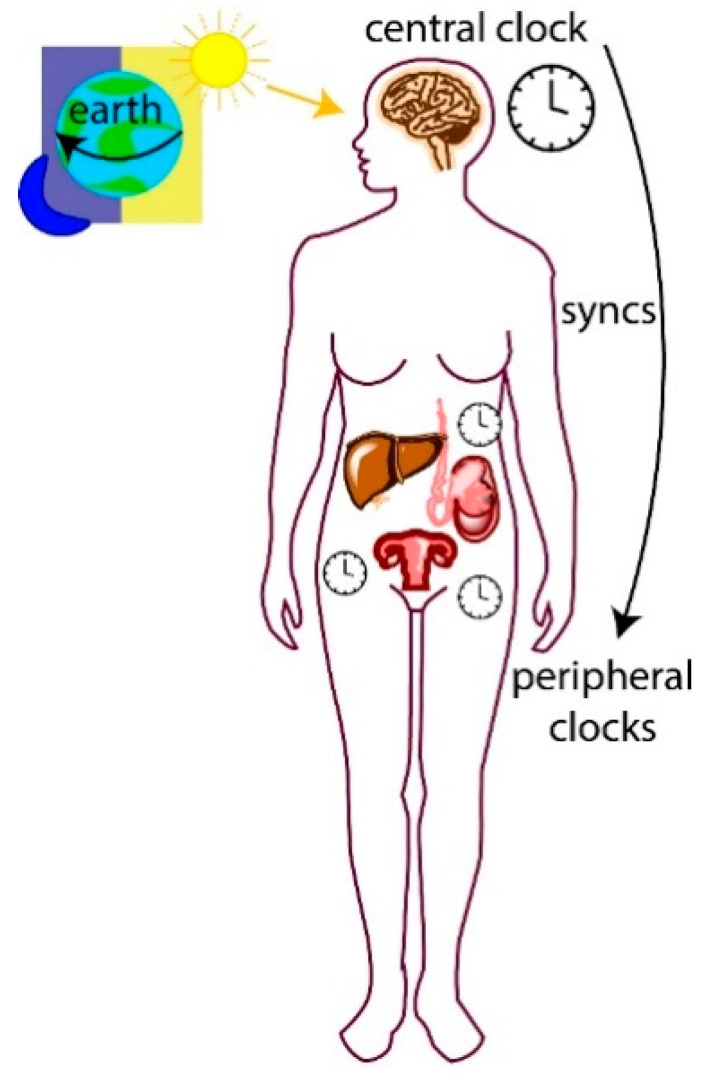
The 24-h rotation of the Earth generates daily cycles of light and dark periods. Mammals have behavioral and physiological changes (including sleep–wake cycles) that correspond to these light and dark periods. Light is detected by photoreceptors in the retina, which relay the signal to the central clock located in the suprachiasmatic nucleus in the hypothalamus. The central clock sends signals to peripheral clocks to ensure all cells within a singular organ are synchronized with each other and with the external time.

The circadian system is hierarchical, and the central clock is composed of a biological pacemaker located in the brain’s suprachiasmatic nucleus (SCN). Peripheral clocks are present in nearly all other tissues of the body and their cellular clocks are synchronized by signals from the central clock (Figure 1). A major signal for synchronization is the daily light–dark cycle detected by photoreceptors in the mammalian retina, which relay signals to the SCN [18]. This signal generates electrical and endocrine signals that are sent to organs and tissues in the body (peripheral clocks) via the autonomic nervous and endocrine systems, leading to synchronization [19,20]. Peripheral cellular clocks also respond to other stimuli including temperature shock, glucocorticoids and timed feeding cues [21,22,23]. For example, in mice, restricted feeding during the daytime uncouples circadian liver gene expression from that in the SCN [24]. Cellular clocks are autonomous and when removed from an organism for cell culture, will continue to oscillate [25,26]. However, without external cues or from a central clock, they lose synchronization with one another.

### 1.2. Molecular Regulation of the Circadian Clock

**Figure 2:**
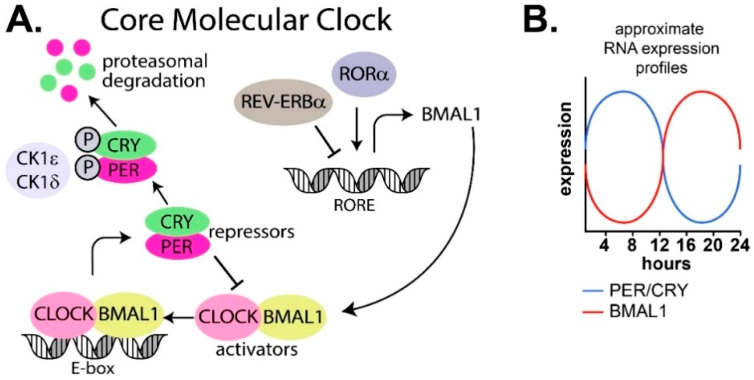
(**A**) The core molecular clock consists of a positive loop (transcription factors BMAL1 and CLOCK), and a negative loop (period—PER and cryptochrome—CRY). BMAL1 and CLOCK heterodimerize and bind to the E-box present in the promoter region of target genes, resulting in expression of PER and CRY. PER and CRY heterodimerize to repress BMAL1 and CLOCK expression. A second feedback loop includes RORα and REV-ERBα, which further control rhythms. RORα increases expression of BMAL1, while REV-ERBα represses BMAL1 expression. The core clock proteins are further controlled by kinases like CK1δ and CK1ε, which can phosphorylate CRY and PER, directing them to ubiquitin-mediated proteasomal degradation (reviewed in [27,41]). (**B**) The transcription–translation feedback loop shown in (**A**) leads to cycling transcriptional profile patterns shown here as relative mRNA levels for PER, CRY and BMAL1 over ~24 h period.

The oscillatory patterns in the SCN and peripheral tissues are generated by a transcription–translation feedback loop present in cells (reviewed in [27]). At its core, the mammalian cellular circadian machinery consists of two interlocking transcription–translation negative feedback loops of genes and proteins that oscillate over a period of 24 h (Figure 2A,B).

The core circadian feedback loop consists of a positive loop of transcription factors BMAL1 and CLOCK, and a negative loop consisting of PER (period) and CRY (cryptochrome). There are multiple isoforms of both PER (PER1/2/3) and CRY (CRY1/2; Figure 2A). BMAL1 and CLOCK heterodimerize and bind to E-box DNA elements in the promoter region of target genes, resulting in expression of period (PER) and cryptochrome (CRY) genes and accumulation of their respective proteins, PER and CRY. PER and CRY enter the nucleus and heterodimerize to repress BMAL1 and CLOCK expression before a threshold level is reached that triggers the cycle to recommence [28,29,30,31,32,33,34,35,36]. PER and CRY are also phosphorylated by the serine/threonine protein kinases, casein kinase 1 delta (CK1δ) and casein kinase 1 epsilon (CK1ε), which marks them for ubiquitin dependent proteasomal degradation [37,38]. This core loop is also influenced by an extensive web of other genes and proteins, including REV-ERBα and RORα, which repress and activate transcription of BMAL1 and CLOCK, respectively [39,40].

## 2. Environmental Disruption of Circadian Rhythms and Links to Cancer

### 2.1. Epidemiological Studies Link Shift Work and Disrupted Circadian Rhythms to Cancer Incidence

An environmental circadian disruption occurs due to a desynchronization between the internal clock and external time, which can occur due to shift work or moving time zones. Epidemiological studies have linked circadian disruption from shift work to an increased incidence of cancer. Nurses who work night shifts have been reported to have higher rates of breast, colorectal, rectal and endometrial cancers [12,42,43,44,45], and this risk increases as the duration of shift work increases [12,44,46]. Women who predominantly work at night also have increased breast cancer risk [47,48,49]. A meta-analysis identified that pre-menopausal women (but not post-menopausal women) had a higher breast cancer risk if they were current or recent night shift workers [50]. Susceptibility to the carcinogenic effects of shift work may be genetic. In one study, night time work increased the risk of breast cancer in women with a specific heterozygotic *CRY2* genotype or who carried at least one particular allele of *RORA*, indicating a genetic interaction with night work in the development of breast cancer [51]. Increased risk of prostate cancer has also been observed in male nightshift workers, and aggressiveness of the disease was found to correlate with the duration of the nightshift [13]. The mounting evidence has prompted the International Agency for Research on Cancer (IARC) to classify shift work as a Category 2A human carcinogen [52].

Disruption of regular sleep schedules also has further detrimental effects. In a prospective cohort study examining the link between work schedule and the development of prostate cancer, male subjects whose occupations involved rotating shifts were at a significantly higher risk of prostate cancer when compared to fixed night workers as well as day workers [53]. Female subjects that do not sleep from 1.00 to 2.00 a.m. have an elevated risk of breast cancer [54].

Studies have also observed higher rates of cancer in subjects with occupations in air travel, which is associated with jetlag. A meta-analysis showed that female flight attendants who have regularly experienced disturbances to their endogenous circadian rhythms exhibit an increased risk of breast cancer and malignant melanoma [55]. Canadian and Norwegian pilots have an elevated incidence of prostate cancer when compared to their respective average population rates [56,57]. However, there may be confounding factors to consider in these studies as airplane travel involves exposure to carcinogenic factors like ionizing radiation, and night shift workers have less exposure to sunlight, which may influence outcomes [55]. For example, nurses who work night shifts have decreased melanoma rates [58]. This lowered risk may occur through mechanisms separate to circadian disruption, as nighttime workers probably have lower exposure to UV radiation. Further investigation is required.

Circadian disruption and its link to carcinogenesis is not merely confined to light exposure during the night, but also extends to meal timing. In a large cohort study of French participants, there was an association between late consumption of the final meal and the risk of developing breast or prostate cancer, further suggesting that disruption of the biological clock can influence the initiation of cancer [59]. Another study also found that a long interval between the last meal and sleep was associated with a lower risk of breast and prostate cancer [60]. It is interesting that most studies of environmental circadian disruption and cancer are around breast and prostate cancer, hinting at a possible hormonal link. However, breast and prostate cancers are also the most common cancers and knowledge on circadian disruption in other cancers may be limited by their comparatively smaller cohort sizes. Further epidemiological data combined with molecular analyses is required to understand the extent of circadian perturbation on carcinogenesis and any role hormones may play.

### 2.2. Animal Models and In Vitro Studies of Circadian Disruption Support the Epidemiological Studies and Demonstrate Increased Tumor Incidence and Faster Tumor Growth

Animal models of environmental circadian disruption commonly mimic the effects of shift work, jet lag and nighttime exposure to light. These models support the clinical observations that environmental circadian disruption can increase tumorigenesis and increase progression and growth of pre-existing tumors. Exposing mice to constant light increases the incidence of lung and liver tumors and leukemias compared to mice under 12 h light:dark cycles [61]. On a similar note, mice who are genetically predisposed to breast cancer and exposed to an inverted light-dark cycle have increased rates of tumorigenesis and more rapid disease progression [62]. They were also more likely to gain weight, implying a link between circadian disruption and metabolism [62]. Introduction of lesions into the mouse suprachiasmatic nucleus disrupts signaling from the central clock to peripheral clocks and results in faster growth of pre-existing tumors and a decrease in survival [63]. In a mouse model of chronic jetlag, the progression of Glasgow osteosarcoma was accelerated in comparison to mice experiencing normal circadian rhythmicity [64]. This finding was also evident in a lung cancer model where jetlagged mice displayed greater malignant transformation, increased tumor growth and increased progression [10].

In vitro experiments support the link between rhythms and growth. Cells in culture maintain individual rhythms but typically lose synchronization with one another due to a lack of resetting cues [25]. Cells can be synchronized with one another using serum [65], heat shock [66], dexamethasone [67] or Forskolin treatment [68]. Restoring circadian rhythms in melanoma and colon cancer cell lines decreases proliferation [69]. When mice are injected with melanoma cells and tumors are allowed to form, the cells within the tumor lose synchronization with one another, despite robust oscillation (and synchronization) in adjacent healthy cells [69]. Resetting cues, such as systemic dexamethasone or timed feeding, resynchronizes rhythms in tumors and decreases growth [69,70].

Genetic manipulation of clock genes also disrupts circadian rhythms in a tissue-specific manner. Experimental deletion of *BMAL1* and *PER2* in the entire murine body resulted in an increase in lung tumor incidence [10]. Deletion of *BMAL1* and *PER2* in cultured cells increases proliferation [10]. *PER2* is a key regulator of the cellular clock and it appears to be particularly important in the occurrence, development and progression of cancer [71,72]. Mice deficient in *PER1* and *PER2* have an increased incidence of lymphomas and a reduced rate of apoptosis following γ-radiation [71]. *PER2* deficient mice also had deregulated cell cycle and a lack of tumor suppression, suggesting that *PER2* functions to suppress tumors through DNA damage-responsive pathways [71]. *PER2* mutations in mouse liver resulted in a fourfold increase in liver cancers [73]. Restoring *PER2* in the tumor cells of a mouse model of sarcoma suppressed tumor growth [74].

## 3. Genetic Disruption of Circadian Rhythms and Links to Cancer

### Mutations, Epigenetic Changes and Deregulated Expression of Clock-Related Genes are Common in Tumor Cells

Cancers occasionally have mutations in clock genes and they commonly have deregulated expression of clock genes. Despite the frequency of clock irregularities, it is still unclear if clock mutations and/or deregulated clock expression can cause cancer. One study systematically analyzed the alterations of clock genes across 32 cancer types using data from The Cancer Genome Atlas (TCGA), Cancer Therapeutics Response Portal and The Genomics of Drug Sensitivity in Cancer databases [11]. The authors found widespread alterations of clock genes at the genetic (mutations), transcriptional (increased and decreased expression) and epigenetic (methylation) levels, which were linked to disrupted circadian rhythms in patient tumor samples. Furthermore, the transcriptional dysregulation of clock genes was associated with patient survival, tumor stage and subtype. The authors concluded that some clock genes function as oncogenes (*ARNTL2*, *NR1D1* and *NPAS2*), while others may act as tumor suppressors (*PERs*, *CRYs* and *RORs*) [11]. A large genome-wide association study of 17 circadian genes found that genetic variation of single nucleotide polymorphisms (SNPs) in 15 of the genes were significantly associated with the risk of cancer further supporting the hypothesis that circadian genes may act as tumor suppressors and/or oncogenes [75].

Isoforms of period were also commonly downregulated in patient tumor cells. Of the 14 cancer types analyzed for gene expression, 11 types had downregulated *PER1* expression, seven types had downregulated *PER2* expression and 10 had downregulated *PER3* expression [11]. Disrupted expression of these *PER* genes correlated with inhibition of apoptosis and increased oncogenic signaling, suggesting an important role of *PER* in cell cycle regulation [11]. Decreased expression of *PER1* and *PER2* has also been observed in gliomas [76,77], pancreatic cancers [78] and breast cancers [79]. One study suggested that decreased expression of *PER1* and *PER2* in breast tumors was due to the methylation of the *PER* gene promoters [79]. Both sporadic and familial breast tumors have decreased expression levels of *PER1* and *PER2* when compared to normal breast tissue [80]. The familial tumors had significantly decreased levels of *PER1* even when compared to sporadic breast tumors, suggesting that aberrant clock gene expression may be important in the development of familial breast cancer [80]. These results support the idea that PERs may act as tumor suppressors.

Ovarian tumors have been found to have decreased expression of *PER1/2*, *CRY2*, *CLOCK*, *CKIε* and *BMAL1* as compared to healthy ovarian tissue [81]. Decreased expression of *PER1/2/3* (and *CRY1/2* and *BMAL1*) has also been observed in the blood of patients with chronic myeloid leukemia (CML) when compared to the blood of healthy individuals [82]. Downregulated expression of *PER3* in the CML tumors is due to inactivation by methylation rather than due to mutation [82], similar to breast tumors [79]. Methylation also silences other clock genes. For example, both leukemia and lymphoma cells have been found to have transcriptionally silenced BMAL1 through promoter CpG island hypermethylation [83]. Restoring *BMAL1* levels in hypermethylated lymphoma/leukemia cells results in growth inhibition [83]. In a study of nurses with breast cancer, exposure to night work was associated with increased methylation of the *CLOCK*, *BMAL1*, *PER1* and *CRY1* genes, compared with controls, suggesting that epigenetic regulation of these clock genes may have a role in breast cancers linked to shift workers [84]. Mutations also play a role in these cancer types. Three SNPs in CRY2 are linked to an increased risk of non-Hodgkin’s lymphoma [85].

Dysfunctional rhythms due to improper clock-related gene expression are linked to worse outcomes in melanoma patients [86] and colorectal cancer patients [87]. The *CLOCK* gene itself is mutated in 53% of colorectal cancer samples [16]. In chronic lymphocytic leukemia, the ratio of *PER2* to *CRY1* is suggested to be a prognostic marker that predicts survival outcomes of patients, with a low *PER2*:*CRY1* having the best outcomes [88]. Furthermore, increased TNF in Hodgkin lymphoma cells has been found to alter core-clock gene expression and cell cycle phase, impacting cell proliferation and migration [89].

**Figure 3:**
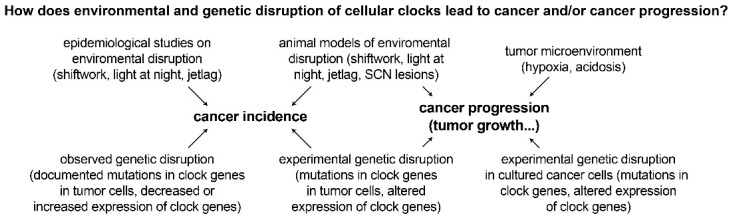
Evidence linking circadian disruption to cancer. Multi-level circadian disruption has been linked to increased cancer incidence and increased cancer progression.

Recent studies have also found that splicing-related genes have temporal expression patterns that oscillate every 24 h [90,91,92]. Alternative splicing of U2-auxiliary-factor 26 has been shown to destabilize PER1 in mice [90]. Aberrant splicing of circadian genes is likely to affect the outcome of splicing events in their target genes, resulting in the production of isoforms that may confer oncogenic properties to cells [91]. Rhythmic isoform expression patterns are tissue dependent and differ between primary tumors and metastatic tumors, suggesting a functional role of rhythmic splicing that may be implicated in tumor progression [91]. The prevalence of mutations in clock genes and aberrant expression of clock genes in various cancers supports the link between circadian disruption and cancer development. Disruption of the molecular clock appears to be a common feature of tumor cells. Furthermore, experimental data is emerging indicating that these genetically dysregulated rhythms play a mechanistic role in multiple cancer steps, including carcinogenesis and tumor progression (Figure 3). The reverse also appears to occur, with common cancer characteristics capable of disrupting the molecular clock.

## 4. Hallmarks and Characteristics of Cancer and their Interactions with Circadian Rhythms

### 4.1. Disruption of Tumor Circadian Rhythms by Microenvironmental Factors Such as Hypoxia and Acidosis

Whilst tumor circadian disruption can be caused by genetic mutations and possibly environmental factors like shift work, alterations to the tumor microenvironment may also contribute. Solid tumors contain rapidly proliferating cells that outgrow the surrounding vasculature. A lack of access to circadian resetting cues in the blood could disrupt tumor cell clocks. For example, melatonin secreted from the pineal gland and insulin secreted from the pancreas both travel in the blood to reset peripheral clocks [93,94], and a lack of tumor vasculature may desynchronize tumor cells from surrounding tissue.

**Figure 4:**
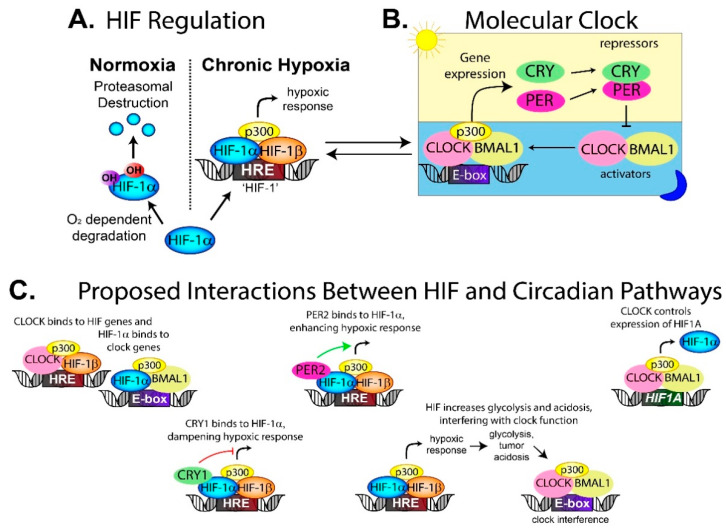
Interactions between the hypoxia inducible factor (HIF) and circadian pathways. (**A**) Under normal oxygen conditions, HIF-1α is degraded through an oxygen-dependent degradation pathway. In hypoxic conditions, like that in a tumor, HIF-1α is no longer degraded and translocates into the nucleus where it binds to HIF-1β and p300 to form the HIF complex. The complex binds to the hypoxia response elements (HRE) in the promoter region of >1000 genes to generate the hypoxic response. (**B**) Simplified version of the molecular clock. For further details see Figure 2. (**C**) Several interactions between the HIF and circadian pathways have been proposed. Given that tumors are commonly hypoxic with disrupted circadian rhythms, it is possible that HIF activation may be interfering with clock gene expression, generating the disrupted rhythms. Arrows indicate activation or increased expression, while T-arrows indicate blocking activity. Parts of the figure modified from [97].

The blood supply also delivers oxygen, and without it, the tumor develops regions of hypoxia, which is correlated with a poor patient prognosis [95]. Hypoxia activates the hypoxia inducible factor (HIF) transcription factor [96], which controls the expression of genes involved in tumor growth, invasion and glycolysis [97,98]. In the canonical HIF pathway, the HIF-1α subunit is degraded in an oxygen-dependent manner in normoxia through the proteasome. In hypoxia, HIF-1α dimerizes with HIF-1β and p300 to form a stable HIF complex that can bind to the hypoxia response elements (HRE) in the promoter region of target genes [97] (Figure 4A). Several studies, including one in hepatocellular carcinoma, have shown that experimentally induced hypoxia alters circadian gene expression in a HIF-1α-dependent mechanism [99].

Clock proteins and HIF proteins can interact and alter downstream transcriptional pathways. CRY1 can bind directly to HIF-1α (and the HIF-2α isoform), which decreases HIF binding to target genes [100] (Figure 4C). Loss of CRY1 in cells increases HIF activity, which increases proliferation and invasion [100]. Separately, PER2 has been found to interact with HIF-1α and enhance HIF-1 activity [101], further demonstrating how circadian proteins can modulate the hypoxic response and therefore alter tumor behavior.

The relationship between HIF and circadian clocks appears to be bidirectional, meaning that HIF can impact on circadian regulation, while components of the circadian clock can affect the HIF hypoxic response. Under normal circumstances, the transcription factor HIF binds to the HRE sites near HIF-target genes (Figure 4A), while BMAL1/CLOCK binds to the E-box located near clock genes (Figure 4B). In contrast, several studies have found HIF-1α co-occupied loci with BMAL1 on core circadian genes, thereby regulating the clock (Figure 4C) [102,103]. Circadian proteins also bound to the *HIF1A* promoter, thus controlling expression of the HIF-1α protein (Figure 4C). This means the clock can fine-tune the hypoxic response depending on the time of day, while HIF-1α can interact with BMAL1 to influence circadian rhythm [102,103].

Physiological oxygen levels have been proposed as a resetting cue for circadian rhythms through HIF-1α. Blood and tissue oxygen levels have been found to have daily rhythms in rodents [104]. In cultured cells, mimicking these physiological oxygen cycles synchronizes cellular clocks in a HIF-1α-dependent manner [104]. Circadian-regulated gene expression was also altered in response to changes in oxygen levels and this was mediated by HIF-1α [104]. Studies in zebrafish also support a bidirectional relationship between the hypoxic response pathway and the circadian clock. HIF-1α bound to the *period1* gene, dampening *period1* oscillation in hypoxic zebrafish larvae [105,106].

Others have suggested that hypoxia disrupts circadian rhythm through HIF-driven changes to the tumor microenvironment instead of through HIF and circadian crosstalk on the genome level. In hypoxia, cells change from oxygen-dependent energy synthesis to glycolysis leading to rapid acidification of the tumor microenvironment, which is HIF-dependent. HIF-1 driven acidification has been proposed as a circadian clock disruptor [17,107,108] (Figure 4C). Decreasing the pH in vitro suppresses cellular circadian oscillation, while buffering media to a neutral pH restores rhythmicity [17]. The low pH seems to dampen circadian oscillations rather than desynchronize [17]. Decreasing the ambient carbon dioxide concentration from 5% to 1% phase-shifted the circadian rhythmicity of BMAL1 in NIH3T3 cells by 5 h [108]. It was proposed that the shift could be caused by a change in pH, or as a result of carbon dioxide directly affecting the circadian clock, which has been previously hypothesized [109].

One interesting question that has emerged from these studies is how the molecular clock may be affected in patients that have both obstructive sleep apnea (OSA) and cancer. OSA causes several physiological stresses including intermittent hypoxia, hypercapnia (increased CO_2_ and acidosis) and circadian disruption due to disrupted breathing and frequent waking. A number of epidemiological studies have linked OSA to worse cancer outcomes and more aggressive tumors (reviewed in [97]). Patients with OSA also have higher rates of cancer [97]. Intermittent hypoxia mimicking sleep apnea has been shown to increase HIF-1α in cultured colorectal cancer cells [110], and it seems feasible that OSA might cause both environmental circadian disruption (frequent arousal during sleep) and molecular circadian disruption (activation of HIF-1α, which can alter the molecular clock), which may negatively affect cancer outcomes. Further work will be needed to determine if this is occurring in vivo and this may alter cancer biology.

While not all of the described hypoxia and circadian studies were in a cancer context [102,103,104], they demonstrate an interesting relationship between hypoxia, which is common in tumors, and circadian regulation, which is commonly disrupted in tumor cells. The idea that the tumor microenvironment can disrupt circadian oscillation is supported by a study showing that healthy skin cells adjacent to a cutaneous melanoma had significantly reduced expression of clock genes [111]. Coculture experiments showed that the cancer cells circadian phenotype, metabolism and survival are affected by surrounding cells [112]. This implies something in the surrounding environment is disrupting the clocks of healthy cells, whether it is through hypoxia, acidosis or secreted factors. Clocks in healthy tissues far from the original tumor, like the liver, have also been found to be reset by lung, breast and skin tumors, possibly through secreted cytokines [111,113,114]. Given that tumor cells can be out-of-sync with each other and with surrounding healthy tissue and/or can cause healthy tissue to become desynchronized, there are likely to be multiple mechanisms driving cellular desynchronization, including microenvironmental effects.

### 4.2. Disrupted Cell Cycles Can Be Controlled by Circadian Rhythms

Another hallmark of cancer is uncontrolled cell proliferation. Cell division and proliferation is controlled by the cell cycle, and progression through the phases of the cell cycle relies on the sequential activation of cyclin-dependent kinases (CDKs), which form a complex with cyclins, triggering specifically timed events. Like circadian rhythms, the cell cycle acts in an oscillatory manner and consists of four phases: G1, S (where DNA replication occurs), G2 and M phase (where cell division occurs). Cells that have temporarily stopped dividing enter the resting G0 phase (reviewed in [115,116]). Entry and exit through phase checkpoints of the cell cycle can be gated by circadian rhythms, which restricts uncontrolled proliferation. In single cell organisms, such as cyanobacteria and flagellate algae, cell division can only occur at specific points in the circadian cycle [117,118,119,120].

In multicellular organisms, the relationship between circadian rhythms and cell cycle is less clear, but there are several documented links. Rhythmic patterns of DNA replication and rhythmic expression of cell cycle proteins have been observed in various human and mouse tissues, including rectal mucosa [121], the alimentary tract, corneal epithelium and bone marrow [122,123], hair follicles [124] and the gastrointestinal tract and skin [125,126]. Stem cell division in the blood, brain and intestine is also cyclical with the molecular clock [127,128,129,130]. In zebrafish, light cycles can directly regulate the timing of the S phase in the skin, heart and gut [131]. The circadian clock has also been found to regulate the cell cycle at the G1/S transition and the G2/M transition in other multicellular organisms [2,5,131,132,133,134,135]. However, it is not entirely clear how varying rates of cellular proliferation can be controlled by a regularly timed circadian cycle. Instinctually, one would imagine that if cell cycle was directly controlled by a highly regular pattern of circadian cycles, then cell division would also be highly regular and follow a similarly timed oscillation (1:1 pattern). This is not always the case and the rate of proliferation varies significantly between tissues. In adult organisms, cells of the brain, liver and heart have a low proliferation rate and mainly exist in the quiescent G0 stage, while cells of the skin, intestinal epithelium, hematopoietic and immune systems are constantly proliferating [115]. However, a change in conditions can increase cell division in the quiescent tissues, which is regulated through circadian rhythms. Neurogenesis of the adult hippocampus is regulated by circadian rhythms through gating at the cell cycle entry and exit [129]. Proliferation rates of cells are also altered by disease state, for example, partial removal of the liver causes hepatocytes to exit the G0 phase and enter the cell cycle in a circadian-dependent manner, regenerating the liver [2]. Similar circadian effects have been observed in wound healing of the skin [136] and intestine [137]. This would imply that dependency of the cell cycle on circadian regulation varies depending on the tissue and environmental changes. There are also examples where cell division does not depend on circadian biology, for example, fibroblasts have rhythmic division cycles, which appear to be independent of circadian oscillation [138]. There are likely to be significant differences in how various tissues regulate proliferation through the cell cycle and further work is needed to understand these nuances.

Many of these studies observed a role for circadian rhythms at the entry and exit points of the cell cycle (‘circadian gating’) in zebrafish [132] and other organisms. This is probably because the circadian clock controls the expression of key cell cycle checkpoints, providing windows in time where the cell must commit to entering the cell cycle or the opportunity will be lost until the next opportunity is offered by the circadian cycle. Expression of the cell cycle regulators, Ccnb1, Ccnd1 (Cyclins B1 and D1), c-Myc, p20 and p21 have been found to be under circadian control (reviewed in [139]). Wee1 transcription can be activated by CLOCK/BMAL1 and is also repressed by PER/CRY [2]. PER1 appears to stabilize c-MYC, suppressing the expression of p21 [7]. PER1 also alters Ccnb1, Cdc2 and Wee1 transcription and expression, which requires p53 [7]. A loss of PER1 in tumor cells therefore removes a regulatory component of the cell cycle [7]. The relationship between cell cycle and circadian rhythms can also behave bidirectionally, for example, cyclin-dependent kinase 9 (CDK9) can modulate the clock by attenuating REV-ERBα activity [140].

Interestingly, mice with mutated circadian clocks still develop normally and are viable indicating cell cycles can occur without a functioning clock (reviewed in [141]). More work is needed to understand the tissue specific differences of circadian control of the cell cycle and the circumstances under which the two become linked. However, given that the cell cycle appears to be under circadian control for at least some tissues, part of the time, it seems possible that cancer cells could increase their cell division and proliferation through disruption of the circadian clock.

### 4.3. Circadian Control of Metabolism

An altered state of metabolism is another characteristic of cancer cells and the regulation of catabolic and anabolic metabolism in different organs is known to be coordinated by circadian rhythms [142]. One study found that a mouse model of chronic jet lag caused metabolic changes in the liver that led to liver cancer [143]. Chronic jet lag led to non-alcoholic fatty liver disease through metabolic disruption in the liver, which progressed to steatohepatitis and fibrosis before eventually causing hepatocellular carcinoma [143]. Rats exposed to constant light rather than normal light:dark cycles have larger tumors and they also become overweight with elevated plasma triglycerides and glucose levels [144]. The increase in tumor volume is thought to be driven by the metabolic changes induced by constant light [144].

Regulation of cellular metabolism is also coordinated by circadian rhythms. Tumors commonly have increased levels of glycolytic metabolism, known as the Warburg effect [145]. Increased glycolysis can be driven by hypoxia and HIF-1α as discussed in Section 4.1, and it can also be driven by the PI3K/AKT and mTOR pathways, which also show circadian fluctuations in their protein levels [146]. mTOR is rhythmically ubiquitylated and this rhythmic expression can also feed back into the core circadian machinery [147]. Activation of AKT has also been found to act on the molecular clock, lengthening the circadian period [148]. AKT-driven phosphorylation of CLOCK directs it for nuclear translocation, affecting the expression of downstream metabolic genes [149]. A number of metabolites display circadian oscillation [150,151,152] and important regulators of metabolism, such as Ras, c-Myc, nuclear hormone receptors and p53 are also associated with circadian rhythms (reviewed in [142,153,154]). Dysfunctional or absent rhythms could potentially deregulate tumor metabolism and alter cancer progression.

### 4.4. Circadian Control of Other Cancer Characteristics

Many deregulated cancer processes are linked to circadian rhythms, including the DNA damage response, DNA repair, immune surveillance and inflammation. DNA repair genes show circadian rhythms in mRNA and protein expression [155,156]. PERs and CRYs have been shown to interact with proteins in the DNA damage response pathways, meaning that there may be daily rhythms in the ability of a cell to sense and repair damaged DNA [142]. An interesting observation is that many of these links are bidirectional. For example, CLOCK/BMAL1 can express the DNA repair gene Xpa in a rhythmic fashion [157], and DNA damage itself can lead to changes in the phase of the circadian rhythm [158,159].

Suppression of the immune system is important for cancer development and progression as it allows cancer cells to avoid immune surveillance. The molecular clock can act on the immune system in several ways, which are only beginning to be understood. Melanoma patients with high expression levels of BMAL1 have increased T-cell activity, a better response to anti-PD1 immunotherapy and increased overall survival, as compared to patients with low BMAL1 levels [86]. Rodents exposed to a jet lag protocol have altered rhythms of cytokines, which disrupts immune cell activity and increases lung tumor growth [160]. Furthermore, genetic disruption of the molecular clock increases a number of pro-inflammatory and immunosuppressive cytokines (reviewed in [142]), which help cancer cells evade the immune system. CLOCK, BMAL1 and the CRYs can also alter the function of NF-κB, which is a transcription factor that has important roles in the immune system and in inflammation [161,162,163]. The clock can also influence immune cell differentiation, trafficking and function [142,164].

## 5. Restoring the Circadian Clock in Tumor Cells May Be an Effective Anti-Cancer Strategy

Restoring the circadian clock in tumor models can reduce tumor growth and may be an effective treatment for cancer. Timed feeding cues in chronically jetlagged mice restores circadian activity and reduces growth of transplanted Glasgow osteosarcoma and pancreatic adenocarcinoma tumors [70]. Resynchronization of tumor cells using dexamethasone reduces melanoma and colorectal tumor cell proliferation by lowering the number of cells entering the S phase of the cell cycle [69]. Furthermore, dexamethasone treatment in mice reduced melanoma tumor growth by 60% [69]. These studies support the idea that circadian resynchronization of tumor cells has anti-cancer effects.

### 5.1. Circadian Anti-Cancer Drug Targets

A number of circadian drug target candidates have emerged, including the REV-ERBs, casein kinases and others. Targeting the key circadian regulators REV-ERBs with small molecules was recently investigated for anti-cancer purposes [165]. REV-ERB agonists SR9009 and SR9011 reset tumor circadian rhythms and reduced glioblastoma growth in mice [165]. Furthermore, these drugs were specifically lethal to cancer cells, while having no effect on the viability of normal cells [165]. Inhibition of REV-ERBβ with the ligand ARN5187 showed anti-proliferative activity in cancer cells [166]. Restoration of BMAL1 and CLOCK circadian oscillation in lymphoma cells by using a PERK (protein kinase RNA-like endoplasmic reticulum kinase) inhibitor reduced tumor cell survival [167].

Several high throughput or chemical screens in cells have identified drugs that can modulate circadian rhythms. These drugs were found to act on CK1ε [168], CK1δ [169], glycogen synthase kinase-3β (GSK-3β) [170] and CK1α [171]. Whether these drugs are effective in cancer has yet to be examined. Casein kinase 2 (CK2) was also recently identified as a regulator of circadian rhythms. Inhibition of its activity lengthened the circadian period and inhibited cancer cell growth [172].

In addition to drugs that directly target the core circadian machinery, inhibiting anti-cancer targets like HIF-1α may impact circadian machinery as discussed in Section 4. There are several ongoing efforts, including our own, to develop HIF inhibitors for cancer treatment. Many of the drugs are aimed at blocking the HIF-1α or HIF-2α subunit from binding to partners such as p300 [173,174,175,176,177,178,179], HIF-1β [180,181,182,183,184,185], HSP90 [186,187] or binding to the DNA itself [188]. p300 also binds to the CLOCK/BMAL1 heterodimer [189] and p300 inhibitors used to target HIF may also target circadian regulation. Given the significant number of cellular processes regulated by circadian rhythms, it is likely that current chemotherapy drugs already impact the clock, albeit in different ways. This leads into the idea of cancer chronotherapy, which will be discussed in the following section.

### 5.2. Timing of Current Chemotherapy Protocols May Improve Efficacy through Circadian Mechanisms

Timed delivery, or chronotherapy, of already established anti-cancer drugs has the potential to increase their efficacy and reduce toxicity, as the circadian clock controls processes that influence drug pharmacokinetics and pharmacodynamics [190]. In addition, many chemotherapy drugs target the cell cycle, which is controlled by circadian rhythms. If rhythms are altered in cancer cells, they may be proliferating at a different time-of-day than healthy tissues. It may be possible to administer chemotherapy to target the proliferating cancer cells while reducing toxicity and minimizing effects on healthy tissue. For example, timed delivery of 5-fluorouracil (5-FU) to colorectal cancer patients decreased mucosal toxicity by five-fold compared to constant delivery [191]. Chronomodulated hepatic arterial infusion of 5-FU was also effective in another study involving patients with colorectal cancer who had been pre-treated with other ineffective chemotherapy agents [192]. Cyclin dependent kinase 4/6 (CDK4/6) inhibitors have also showed similar trends, for example, PD0332991, which is a clinically approved anti-cancer agent, was shown to reduce tumor growth in mice in a time-of-day–specific manner [193]. The drug was more effective when administered in the morning compared to the night due to circadian regulation of the G1/S phase transition of the cell cycle [193]. Daytime rather than nighttime administration of seliciclib, a cyclin-dependent kinase inhibitor, reduced Glasgow osteosarcoma tumor growth and improved circadian gene expression within the tumor [194]. mTOR activity has also been found to have a 24-h rhythmic pattern in cultured breast cancer cells and the efficacy of in vitro everolimus, an mTOR inhibitor, varied depending on the timing of the dose [195]. More research is needed into understanding how current drug protocols may be able to be optimized to target physiological and cellular circadian rhythms and improve cancer outcomes.

### 5.3. Melatonin

Melatonin is a pineal hormone whose synthesis and secretion is largely regulated by light-dark cycles [196]. Melatonin acts as a cue that can regulate physiological rhythms and suppression of melatonin has been proposed to increase breast and endometrial cancer risk (reviewed in [197,198,199]). Further to this, administered melatonin appears to have many anti-tumor effects, including effects on apoptosis, proliferation, invasion, metabolism and the DNA damage response pathway [200,201,202,203]. Melatonin can resynchronize prostate cancer cells by counteracting cancer-related changes in circadian gene expression [204]. Administration of melatonin also reduces metastases in a breast cancer mouse model [205,206]. Co-administration of melatonin with chemotherapy may be an anti-cancer strategy worth investigating, but caution is warranted, as administering melatonin at the wrong time-of-day has also been shown to increase tumor growth or have no effect [197]. There is also conflicting information where melatonin has varying effects depending on the season (time-of-year; reviewed in [197]). Melatonin can impact on metabolism (which is circadian regulated and discussed in Section 4.3). In one study, giving melatonin to chronically jet-lagged mice and obese mice attenuated circadian disruption and promoted adipocyte proliferation [207]. This means that melatonin may be useful to prevent and treat sleep deprivation-caused obesity but further studies are needed. In addition, further studies are required to understand how melatonin affects tumor cells before trials in humans are pursued.

## 6. Conclusions

A functioning circadian clock is required for maintaining physiological homeostasis. While there are epidemiological, clinical and experimental links between cancer and circadian disruption, there is still much to learn about circadian mechanisms of cancer causation and/or progression.

First, there is the effect of environmental circadian disruption, which has been linked to cancer. This includes shift work and nighttime work, jetlag and nighttime light exposure. These effects may be working through the suprachiasmatic nucleus. Perhaps disrupting the central clock has effects on the peripheral clocks, which increase tumorigenesis and/or enhance progression of a pre-existing tumor. Several circadian genes have been proposed to act as oncogenes or tumor suppressors and altered expression could increase tumor formation and/or progression in a context-dependent manner [11]. Circadian disruption also has effects on the immune system and a loss of surveillance due to disrupted rhythms could allow for increased tumor formation [208]. Furthermore, systemic disruption to all cellular clocks may interfere with circadian-regulated processes like DNA repair, increasing mutations (reviewed in [9,209]). Cell cycle and proliferation are also tightly linked to circadian rhythms and disrupted oscillations may increase proliferation.

Second, there is genetic disruption of cellular circadian rhythms, which is also linked to cancer. Mutations in circadian genes occur in tumor cells and this may have effects on cell cycle and growth. Experimental deletion of key circadian genes in animals results in increased tumor incidence and cellular proliferation. Altered expression of circadian genes is commonly detected in tumors and when this is replicated in animals it can increase tumor formation and cancer progression. These genetically altered tumor rhythms are linked to a poor cancer prognosis and worse patient outcomes.

Third, alterations in the tumor microenvironment may also contribute to circadian disruption. Tumors are both hypoxic and acidic and current research into how this impacts circadian gene expression is still being explored. Hypoxia activates HIF, and HIF can act on the molecular clock through several mechanisms. The circadian clock can also act to regulate the HIF hypoxic response, indicating a bidirectional relationship. HIF also controls expression of glycolytic genes, which generate the acidic tumor environment, and acidity is thought to act on the tumor cellular clocks, dampening their rhythms and potentially desyncing them from surrounding tumor cells and more distal healthy tissues.

Restoring normal circadian rhythmicity in tumor models reduces tumor growth. The development of new drugs targeting both the core circadian machinery and HIF-1α is an emerging area of research that may prove a useful anti-cancer strategy. Furthermore, timed administration of already established cancer treatments may increase their efficacy and improve current patient outcomes, suggesting an important role for the circadian cycle in drug delivery. While more work is required to understand how circadian rhythms and cancer interact, they are clearly an important factor that should be considered when studying the causes of cancer and developing new treatments.

## Abbreviations

5-FU	5-Fluorouracil
ARNTL2	Aryl Hydrocarbon Receptor Nuclear Translocator Like 2 (BMAL2)
BMAL1	Brain and Muscle ARNT-like protein-1
Cdkn1a	Cyclin Dependent Kinase Inhibitor 1A
CDK4/6	Cyclin-Dependent Kinase 4/6
ChIP	Chromatin Immunoprecipitation
CML	Chronic Myeloid leukemia
CLOCK	Circadian Locomotor Output Cycles Kaput
CK1δ	Casein Kinase 1 Delta
CK1ε	Casein Kinase 1 Epsilon
CRY1/2	Cryptochrome
DEX	Dexamethasone
GSK-3β	Glycogen Synthase Kinase -3 Beta
HIF	Hypoxia Inducible Factor
HIF-1α	Hypoxia-inducible factor 1-alpha
HRE	Hypoxia Response Element
IARC	International Agency for Research on Cancer
NR1D1	Nuclear receptor subfamily 1, group D, member 1 (gene for REV-ERBα)
NPAS2	Neuronal PAS Domain Protein 2
PER1/2/3	Period1, Period2, Period3
PERK	Protein kinase RNA-like Endoplasmic Reticulum Kinase
ROR	Retinoic acid receptor-related orphan receptor
SCN	Suprachiasmatic nucleus
SNP	Single-nucleotide polymorphism
